# Antioxidant Adjustments of Olive Trees (*Olea Europaea*) under Field Stress Conditions

**DOI:** 10.3390/plants10040684

**Published:** 2021-04-01

**Authors:** Márcia Araújo, João Prada, Nuno Mariz-Ponte, Conceição Santos, José Alberto Pereira, Diana C. G. A. Pinto, Artur M. S. Silva, Maria Celeste Dias

**Affiliations:** 1 Centre for Functional Ecology, Department of Life Sciences, University of Coimbra, Calçada Martim de Freitas, 3000-456 Coimbra, Portugal; marciaaraujo@fc.up.pt; 2 Integrated Biology and Biotechnology Laboratory, LAQV-REQUIMTE, Department of Biology, Faculty of Sciences, University of Porto, Rua Campo Alegre, 4169-007 Porto, Portugal; joaoprada47.jp@gmail.com (J.P.); up201507752@edu.fc.up.pt (N.M.-P.); csantos@fc.up.pt (C.S.); 3 Center for the Research and Technology of Agro-Environmental and Biological Sciences, University of Trás-os-Montes and Alto Douro, 5001-801 Vila Real, Portugal; 4 Centro de Investigação de Montanha (CIMO), ESA, Instituto Politécnico de Bragança, Campus de Santa Apolónia, 5300-253 Bragança, Portugal; jpereira@ipb.pt; 5 LAQV-REQUIMTE, Department of Chemistry, University of Aveiro, 3810-193 Aveiro, Portugal; diana@ua.pt (D.C.G.A.P.); artur.silva@ua.pt (A.M.S.S.)

**Keywords:** climate change, flavonoids, secoiridoids, *Olea europaea*, rainfed olive groves, drought

## Abstract

Extreme climate events are increasingly frequent, and the 2017 summer was particularly critical in the Mediterranean region. Olive is one of the most important species of this region, and these climatic events represent a threat to this culture. However, it remains unclear how olive trees adjust the antioxidant enzymatic system and modulate the metabolite profile under field stress conditions. Leaves from two distinct adjacent areas of an olive orchard, one dry and the other hydrated, were harvested. Tree water status, oxidative stress, antioxidant enzymes, and phenolic and lipophilic metabolite profiles were analyzed. The environmental conditions of the 2017 summer caused a water deficit in olive trees of the dry area, and this low leaf water availability was correlated with the reduction of long-chain alkanes and fatty acids. Hydrogen peroxide (H_2_O_2_) and superoxide radical (O_2_^•–^) levels increased in the trees collected from the dry area, but lipid peroxidation did not augment. The antioxidant response was predominantly marked by guaiacol peroxidase (GPOX) activity that regulates the H_2_O_2_ harmful effect and by the action of flavonoids (luteolin-7-*O*-glucuronide) that may act as reactive oxygen species scavengers. Secoiridoids adjustments may also contribute to stress regulation. This work highlights for the first time the protective role of some metabolite in olive trees under field drought conditions.

## 1. Introduction

The Mediterranean basin is the world’s leading region of olives and olive oil production [1]. However, this area is increasingly facing climate change events characterized by combined extreme weather conditions [2]. The summer of 2017 was particularly critical to this region (including Portugal and Spain) due to prolonged water deficit periods accompanied by heatwaves and high UV-irradiance levels [3].

Olive trees (*Olea europaea*) have outstanding phenotypic plasticity [4]. However, the nature of these climate change-associated extreme episodes and the predictions of their increase in frequency and intensity in the next decade [2] represent a new threat to olive cultivation. This threat may potentially lead to significant losses in olive productivity and fruit and oil quality [4,5,6,7,8,9].

Olive trees have evolved morpho-physiological adaptive mechanisms to cope with the Mediterranean climate [10]. These include an efficient ability to control leaf transpiration (e.g., thick cuticle, high density of foliar tissue, and trichome layers), and to regulate stomata aperture, osmotic adjustment, and extract water from soils with low water potential [4]. However, the investment of olive energy resources (or shifts in metabolic pathways) in defense mechanisms compromise growth and yield [11]. Both water deficit and heat may reduce olive water status, decrease stomata aperture, impair photosynthesis, and lead to oxidative stress [8,12,13,14]. The prevention of cellular oxidative damage (e.g., lipid, protein, pigments, and DNA) is one of the first stress tolerance mechanisms [15]. To control the increase of reactive oxygen species (ROS), olive plants activate the antioxidant battery, upregulating antioxidant enzymes [e.g., superoxide dismutase (SOD), catalase (CAT), glutathione reductase (Gr), ascorbate peroxidase (APX), and guaiacol peroxidase (GPOX)], and invest in the production of several antioxidant metabolites, like ascorbate, glutathione, carotenoids and phenolic compounds [8,14,16,17].

Polyphenols are among the most significant antioxidants [18,19]. For instance, in response to drought or heat, olive plants increase flavonoids in leaves, especially the catechol B-ring substituted flavonoids (e.g., quercetin 3-glycoside), acting as protective antioxidants [8]. Flavonoids can accumulate in the leaf epidermis, acting more efficiently as UV shields, or they can be found near the cell compartments where ROS is generated (or transported to the sites of ROS generation), acting as ROS scavengers [19,20,21]. The secoiridoids, such as oleuropein, are also another group of polyphenols with a high antioxidant activity, which can only be found in the Oleaceae family [22]. This family of compounds, together with flavonoids, has been reported to have a defensive role in olive and other Mediterranean species exposed to salt stress or high UV-B radiation [8,14,23,24]. Moreover, this family of compounds’ abundance and the ratio strongly depend on the growing environmental conditions, species, cultivar, and organ. In addition to these families of metabolites, also lipophilic compounds related to cuticle wax (e.g., long-chain alkanes and ursolic acid) and with ROS scavenger capacity (e.g., thymol glycosides and ursolic acid), together with compounds associated with the maintenance of membrane integrity (e.g., oleic and palmitic acids) were identified and reported to contribute to olive adaptation to high UV-B radiation [7].

Field extreme stressful episodes may trigger physiological and biochemical responses different from those activated by mild stressing conditions [25,26]. Thus, understanding the changes in these metabolites’ profiles under extreme climate change conditions will support the selection of more adapted cultivars and contribute to ensuring olive productivity and olive/oil quality in the following decades. Most studies on young olive plants used single [11,14] or combined (drought and heat) stressful conditions [5,10] imposed in a controlled environment, which is easier to manipulate but does not mimic the complex environmental conditions of the field. Moreover, the source–sink relationships, determinant for photosynthetic acclimation process, in young potted plants are completely distinct from adult field-grown trees. Besides, potted plants in climate chambers or greenhouses are subjected to stronger effects (e.g., high temperatures in low- or non-ventilated greenhouses) and growth conditions are far from what occurs in the field [27]. Thus, it is not reliable to compare potted plants’ responses growing in climate chambers or greenhouse with those obtained under natural field experiments. Therefore, it is necessary to study olive tree stress responses under natural conditions to provide a real scenario of the stress-induced adjustments in trees and their impacts on olive productivity and oil quality. Persistent drought is particularly important in rainfed orchards and, alone or combined with other stressful conditions, causes agricultural production losses worldwide [28].

We hypothesized that adult field olives’ physiological plasticity to stressful conditions in the field involves adjusting lipophilic and phenolic metabolic pathways, and the activation of antioxidant enzymes. Hence, a phenolic and lipophilic metabolite profiling and a throughout redox status analysis were conducted in leaves of *O. europaea* trees from an orchard exposed to drought conditions, as verified in the summer of 2017 in the Mediterranean region.

## 2. Results

### 2.1. Leaf Water Content and Plant Oxidative Stress

The leaf water content (LWC) in the dry area trees was significantly lower than in the hydrated area, showing a percentage of 50.4 ± 0.9 and 95.3 ± 3.6, respectively.

**Figure 1:**
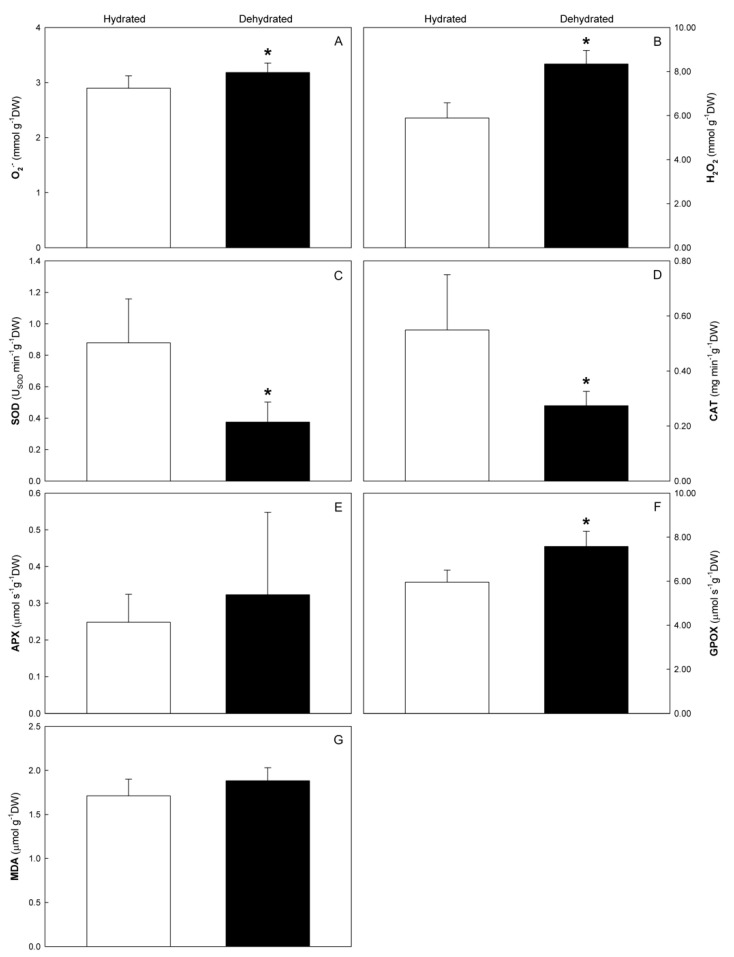
O_2_^•–^ (**A**), H_2_O_2_ (**B**), SOD (**C**), CAT (**D**), APX (**E**) and GPOX (**F**) activities, and MDA (**G**) contents in Cobrançosa’ olive trees leaves from hydrated and dry areas. Values are mean ± standard deviation (*n* = 9). Asterisk (*) indicates significant differences (*p* ≤ 0.05) between conditions.

Regarding oxidative stress, plants from the dry area presented significantly higher values of superoxide radical (O_2_^•–^) and H_2_O_2_ than plants in the hydrated area (Figure 1A,B, respectively). The activity of SOD and CAT was significantly lower in the leaves of trees from the dry area (Figure 1C,D, respectively), and the opposite (increased activity) occurred with the activity of GPOX (Figure 1F). The activity of APX and the MDA content remained similar in both conditions (Figure 1E,G, respectively).

### 2.2. Phenolic Profile

Table 1 showed the phenolic profile and the fold changes in these metabolites. The profile of response of flavonoids and secoiridoids was heterogeneous, as, within each group, both negative and positive responses to the environmental conditions were observed.

Flavonoids were represented in the leaf samples by the quercetin-3-*O*-rutinoside, luteolin, luteolin-7-*O*-glucoside, luteolin-7-*O*-glucuronide, luteolin-4-*O*-glucoside (and its isomer), apigenin-7-*O*-glucoside, and apigenin-7-*O*-hexosyl rhamnosides. Leaves from the dry area showed a level of luteolin-4-*O*-glucoside (and its isomer), luteolin-7-*O*-glucoside, and apigenin-7-*O*-glucoside higher (*p ≤* 0.05) than the ones found in the hydrated area. The levels of apigenin-7-O-hexosyl rhamnosides and quercetin-3-*O*-rutinoside were similar (*p* > 0.05) in leaves from trees of both areas. The luteolin and luteolin-7-*O*-glucuronide levels were lower (*p ≤* 0.05) in leaves from the dry area trees than those from the hydrated area. The secoiridoids were represented by oleoside-11-methyl ester (and its isomer), oleuropein, oleuropein aglycone, and 6’-*O*-[8-hydroxy-2,6-dimethyl-2-octenoyloxy] secologanoside. Leaves from the dry area trees presented the levels of oleoside-11-methyl ester, oleuropein, and oleuropein aglycone higher (*p ≤* 0.05) than those of the hydrated area. Contrarily, the amount of oleoside-11-methyl ester (isomer) and 6’-*O*-[8-hydroxy-2,6-dimethyl-2-octenoyloxy] secologanoside were significantly lower in leaves from the trees in the dry area.

Oleuropein aglycone, oleoside-11-methyl ester (isomer), luteolin-4-*O*-glucoside, luteolin, and luteolin-7-*O*-glucuronide presented a higher amount of differences between leaves from both areas tested. While leaves in the dry area had a higher quantity of oleuropein aglycone and luteolin-4-*O*-glucoside than those found in the hydrated area. Oleoside-11-methyl ester (isomer), luteolin, and luteolin-7-*O*-glucuronide in leaves from the dry area were the ones whose quantities decreased more than those presented in the hydrated area.

### 2.3. Lipophilic Profile

Table 2 showed the lipophilic profile and the fold changes of metabolites in dehydrated leaves concerning the hydrated ones. The profile of response of fatty acids, long-chain alkanes, and sterols was very similar, presenting a negative response characterized by a decrease of these metabolites. Contrarily, the response profile of carbohydrates and terpenes was positive, representing an increase of the respective metabolites.

Fatty acids were represented in the leaf samples by oleic, linoleic, palmitic, stearic, and α-linolenic acids. Leaves from the dehydrated trees presented lower (*p* ≤ 0.05) levels of α-linolenic acid, oleic acid, linoleic acid, and palmitic acid compared to the hydrated ones’ values. Stearic acid presented similar (*p* > 0.05) values in both sampling areas (hydrated vs. dehydrated trees). Terpenes were represented in the leaf samples by squalene, lupeol derivative, ursolic acid, and α- and β-amyrin. These compounds’ levels were similar (*p* > 0.05) in the leaves of trees from hydrated and dry areas. Sorbitol and d-glucose were the only quantified polyol and sugar identified in leaves from trees of the hydrated and dry areas. Leaves from the dehydrated trees presented higher (*p* ≤ 0.05) levels of sorbitol and D-glucose than the hydrated ones. β-Sitosterol was the only sterol identified in olive leaves, and samples from the dry area presented less (*p* ≤ 0.05) levels than samples from the hydrated one. Six long-chain alkanes were identified in the leaves. For the long-chain alkane 1, 3, 4, 5, and 6, dehydrated leaves showed lower (*p* ≤ 0.05) levels than the hydrated ones.

## 3. Discussion

The frequency of climate change events is increasing, and the year 2017 was particularly atypical in several regions of the Mediterranean, with scarce precipitation and prolonged heatwaves. From April to December 2017, Portugal was under severe/extreme drought conditions [29], particularly the region of Trás-os-Montes and Alto Douro, one of the reference regions of olive production. In this region, olive groves are mainly under dryland farming conditions.

**Figure 2:**
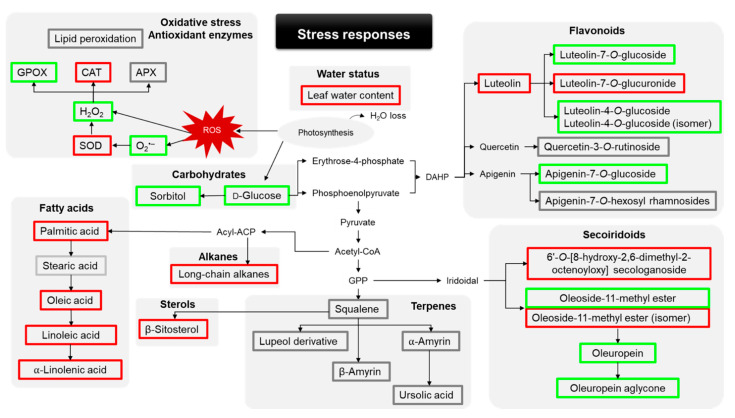
General overview of the responses of olive trees to field conditions. Parameters analyzed are present in boxes surrounded by a green, red, and grey square that indicates a significant increase, decrease, and no significant difference in the abiotic stress response, respectively. Between black arrows occurs multiple reactions. DAHP, 3-deoxy-D-arabino-heptulosonate-7-phosphate; ACP, acyl carrier protein; GPP, geranyl pyrophosphate.

A general overview of the oxidative damages and antioxidant enzymes response, and lipophilic and phenolic profile adjustment occurred in trees from the dry area of the orchard compared to the ones from the hydrated area was presented in Figure 2.

**Figure 3:**
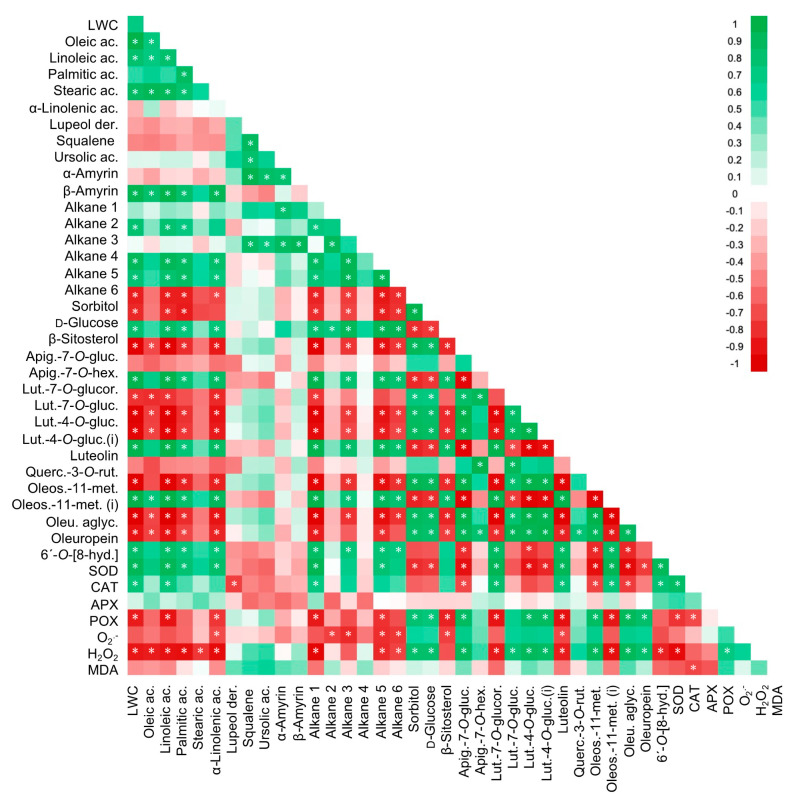
Color rectangles are correlation coefficients; green means a positive correlation coefficient, and red means a negative correlation coefficient. Significant differences (*p* ≤ 0.05) are indicated by a white asterisk (*). Alkane, long-chain alkane; Lut.-7-*O*-gluc., luteolin-7-*O*-glucoside; Lut.-7-*O*-glucor., luteolin-7-*O*-glucuronide; Lut.-4-*O*-gluc., luteolin-4-*O*-glucoside; Lut.-4-*O*-gluc. (i), luteolin-4-*O*-glucoside (isomer); Querc.-3-*O*-rut., quercetin-3-*O*-rutinoside; Apig.-7-*O*-gluc., apigenin-7-*O*-glucoside; Apig.-7-O-hex., apigenin-7-*O*-hexosyl rhamnosides; 6’-*O*-[8-hyd.], 6’-*O*-[8-hydroxy-2,6-dimethyl-2-octenoyloxy] secologanoside; Oleos.-11-met., oleoside-11-methyl ester; Oleos.-11-met. (i), oleoside-11-methyl ester (isomer); Oleu. aglyc., oleuropein aglycone; ac., acid; Lupeol der., lupeol derivative.

Native in the Mediterranean ecosystems, olive trees have evolved characteristics of protection against water loss through tight control of the stomata aperture, a high-density foliar tissue, and thick cuticle and trichome layers [11,30]. However, our study reveals that the absence of precipitation in this region in 2017, coupled with high temperature, induced dehydration symptoms in olive trees, corroborated by the lower leaf water content observed in dry area trees. Interestingly, our data showed that leaf water availability correlated positively with both long-chain alkanes (long-chain alkanes 1, 3, 5 and 6: r = 0.78–0.96, *p* ≤ 0.02, Figure 3) and fatty acids (linoleic acid: r = 0.994, *p* ≤ 0.001; palmitic acid: r = 0.808, *p* = 0.015; α-linolenic acid: r = 0.879, *p* = 0.004; Figure 3), which are main components of the leaf wax cuticle that also confers a barrier to limit water loss [31,32,33]. Thus, the stress conditions decreased the accumulation of some wax cuticle components, lowering water loss protection. Some studies in olive reported that drought tolerance implied an increase of the cuticle thickness [12] and the number of wax components like long-chain alkanes [33]. In line with these findings, we suggest that the orchard’s prolonged exposure to the extreme meteorological conditions of 2017 influenced Cobrançosa trees’ responses, leading to a profile different from Brito et al. (2018) [12]. Furthermore, the decrease of long-chain alkanes and fatty acids in response to the stress conditions can also represent a metabolic shift to promote osmoprotectant agents (e.g., carbohydrates and polyols) or/and antioxidants (e.g., secoiridoids and flavonoids, Figure 3).

The pool of two important osmoprotectant agents, sorbitol (an isomer of mannitol, the main polyol usually found in olive [34]) and D-glucose, increased under the stress condition, a typical response of olive trees to drought and heat stress [5,34]. However, the accumulation of these osmotic agents was not sufficient to maintain cell turgor in trees of the dry area (correlation between LWC vs. D-glucose r = −0.826, *p* = 0.012; and LWC vs. sorbitol r = −0.861, *p* = 0.006; Figure 3). Besides osmolytes, these compounds also represented a carbon and energy source available under stress conditions, and sorbitol seems to act as a defense compound against photo-oxidative damages [7,34].

The stress condition enhances some ROS levels (O_2_^•–^ and the H_2_O_2_) in olive leaves. However, this increase did not induce lipid peroxidation (a key endpoint to cell damage), supporting that this species had a tolerance mechanism to cope with the imposed stress condition [5,7,14]. Some lipophilic compounds’ contribution to membrane protection in Cobrançosa trees exposed to stress also deserves further studies. For instance, despite being lower under stress, the ratio of unsaturated/saturated fatty acids remained similar (hydrated leaves: 1.6 and dehydrated leaves: 1.4), a possible contribution to support the membrane integrity as also suggested by Dias et al. (2018) [7].

The enzymatic and non-enzymatic (e.g., phenolic compounds) antioxidant systems also play an important role in maintaining oxidative damage levels under abiotic stresses [7,11,14]. The antioxidant enzymes are usually the first line of defense against ROS’s excess [35]. In Cobrançosa trees, the lower stimulation of APX and CAT activities under those stress field conditions suggested that H_2_O_2_ is used by GPOX that reduces its putative harmful effect. This is also corroborated by the strong positive correlation between GPOX and H_2_O_2_ (r = 0.714, *p* = 0.047, Figure 3). Contrarily to other works that studied olive plants under controlled environmental conditions and during a short-term stress experiment [14,17], our data showed that SOD activity in Cobrançosa leaves was not stimulated at the moment of sampling (October 2017). Bacelar et al. [36] also reported an upregulation of the GPOX activity in olive trees (*cv.* Cobrançosa) growing under field conditions in response to water deficit, while the SOD activity decreased and the O_2_^•–^ accumulated in leaves resulting in oxidative membrane damages. In turn, Sofo et al. [37,38] showed that in young potted olive plants from the cultivar Corantina, SOD, APX, CAT and GPOX activities increased as the severity of the water deficit increased. Data also show that additional sources of H_2_O_2_ may include the oxidation of fatty acids, glycolate, and the oxidation of xanthine/hypoxanthine to uric acid [17,39,40]. For instance, during the oxidation steps of α-linolenic acid, it occurs the generation of H_2_O_2_ and jasmonic acid [41], a key phytohormone and signaling molecule that regulates abiotic stress responses and modulates plant growth and development [42]. In Cobrançosa, fatty acids decreased in general, particularly the strong negative correlation between α-linolenic acid and H_2_O_2_ (r = −0.922, *p* = 0.001, Figure 3) supports our hypothesis. Concerning the other ROS measured, the excess of O_2_^•–^ generated under stress conditions could be detoxified by other antioxidant compounds [35] and/or via the enzyme SOD and then converted in H_2_O_2_ and used mostly by the enzyme GPOX, as described above.

Besides the referred antioxidant strategies, the role of flavonoids and secoiridoids as antioxidants in olive trees was also very representative [8,14]. The leaf phenolic’s profile of Cobrançosa leaves was very similar to other olive cultivars [14,43]. Within the flavonoids, quercetin-3-*O*-rutinoside, and some luteolin derivatives such as luteolin-4-*O*-glucoside and luteolin-7-*O*-glucuronide, and the secoiridoids oleoside-11-methyl ester and oleuropein were the most representative in Cobrançosa leaves and possibly related to this species’ high tolerance to stress [14,44]. The flavonoids with the catechol B-ring structure, such as luteolin-7-*O*-glucuronide, were recognized as “effective antioxidants” and responded to the stress condition by reducing their levels. This response suggested using these flavonoids into neutralizing radicals and forming flavonoids radicals [18], or the reduction/inactivation of the enzymes that catalyse their synthesis [45]. The oxidative reactions resulting from the flavonoid radicals formed may conjugate with/or stimulate other antioxidant pathways such as the ascorbate/glutathione cycle [46,47]. Considering that flavonoids comprised a wide group of compounds with multiple antioxidant functions, they were distributed by different cells and cellular compartments [46]. For instance, Agati et al. (2013) [20] proposed a mechanism of flavonoids transport from the place of their biosynthesis to several cellular centers where ROS were generated, and/or even the transport of H_2_O_2_ by flavonoids to sites where they were neutralized (e.g., by a system consisting of GPOX/flavonoids/ASC). In olive leaves, the strong correlations between H_2_O_2_ vs. GPOX (r = 0.714, *p* = 0.047, Figure 3) and H_2_O_2_ vs. luteolin-7-*O*-glucuronide (r = −0.877, *p* = 0.004; Figure 3) can also support these findings. Additionally, other flavonoids also changed in Cobrançosa leaves in response to the stress condition. For instance, luteolin pool decrease was correlated to the increase of luteolin-4-*O*-glucoside (r = −0.962, *p* ≤ 0.001, Figure 3) and its isomer (r = −0.913, *p* = 0.002, Figure 3), and luteolin-7-*O*-glucoside (r = −0.708, *p* = 0.049, Figure 3), suggesting that luteolin was used to the synthesis of these derivatives. The accumulation of these luteolin derivatives and the flavonoid apigenin-7-*O*-glucoside possibly represent an improved availability of defense/protective compounds under stress.

Besides flavonoids, one of the main secoiridoids, oleuropein, also had an important antioxidant role, the catechol group in the A-ring had the capacity for scavenging free radicals [48,49]. Similar to that described for other olive cultivars exposed to abiotic stress [11,24], oleuropein accumulation was related in some way to antioxidant availability protection. Other compounds biosynthetically related to oleuropein were also responsive to the stress conditions. For instance, the increase of the oleoside-11-methyl ester pool may result from the oleuropein degradation (r = 0.924, *p* = 0.001, Figure 3) by esterases [50,51]. In addition, the decrease of oleoside-11-methyl ester isomer may represent that it is being used to produce more oleuropein, supporting the increased levels of oleuropein (oleuropein vs. oleoside-11-methyl ester isomer r = −0.876, *p* = 0.004, Figure 3). Other secoiridoids, such as 6’-*O*-[8-hydroxy-2,6-dimethyl-2-octenoyloxy] secologanoside decreased in Cobrançosa leaves under stress, but its role in plants remains unclear, putatively regulating stress response [52].

Our data were in line with other *O. europaea* studies, where the antioxidant pattern of response to abiotic stressors was based on the accumulation of lipophilic protective compounds, activation of antioxidant enzymes, and increase of phenolic compounds [7,8,14,53]. However, it should be considered that the involvement and contribution of specific antioxidant metabolites to plant stress response seems to vary according to the type of abiotic stress and cultivar. For instance, in Galega plants exposed to high UV-B, oleuropein (and its derivatives) play a more critical role than flavonoids and hydroxycinnamic acid derivatives by acting as signaling molecules and ROS scavengers [14]. However, these plants also showed an essential contribution of the enzymes SOD, CAT, GPOX, and APX to maintain membrane integrity [14].

## 4. Materials and Methods

### 4.1. Reagents and Standards

Dithiothreitol (DTT), nitroblue tetrazolium (NBT), H_2_O_2_, titanium dioxide, dichloromethane, pyridine, *N*,*O*-bis(trimethylsilyl) trifluoroacetamide, trimethylsilyl chloride, sodium azide, thiobarbituric acid (TBA), methionine, catalase, peroxidase, cholesterol, maltose, octadecane, phenol, palmitic acid, sorbitol, ammonia and potassium phosphate buffer were purchased from Sigma-Aldrich (Saint Louis, MO, USA). Tetracosane and 4-aminoantipyrine were purchased from Fluka (Bucharest, Romania). Sulfuric acid was purchased from Fisher Chemical (New Hampshire, EUA). Trichloroacetic acid (TCA) was purchased from BIOCHEM Chemopharma (Cosne-Cours-sur-Loire, France). Guaiacol and polyvinylpyrrolidone (PVP) were purchased from Acrós Organics. Triton-X and ascorbic acid were purchased from Panreac. Acetic acid, ethylenediaminetetraacetic acid (EDTA) and phenylmethylsulfonyl fluoride (PMSF) were purchased from VWR (Pennsylvania, EUA). Riboflavin, *n*-hexane p.a., methanol p.a., sodium carbonate (Na_2_CO_3_), dimethyl sulfoxide (DMSO), potassium sulfate and quercetin were purchased from Merck (Darmstadt, Germany). Luteolin and oleuropein were purchased from Extrasynthese (Genay, France).

### 4.2. Plant Material and Field Experiment

**Figure 4:**
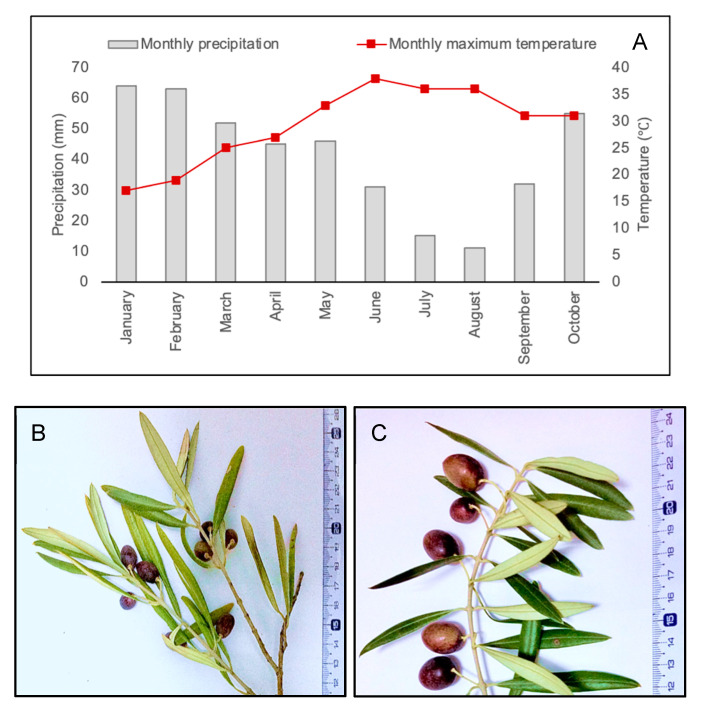
(**A**) Monthly average precipitation, monthly maximum air temperature from January to October of 2017 in the local of the olive orchard. (**B**) Branch of olive trees from the dry area; and (**C**) branch of olive trees from the hydrated area.

The selected rainfed olive grove is located in the northwest of Portugal, Trás-os-Montes region, the producing region of the Protected Designation of Origin “Azeite de Trás-os-Montes”, in Suçães-Mirandela (41°30′39.5″ N 7°15′28.1″ W). The grove consists of adult trees from the cultivar Cobrançosa (one of the most representative cultivars in Portugal) and is about 30 years old. This olive grove is managed as dryland farming, following the integrated production guidelines for olive groves. This orchard is located in a region classified as Csb, according to the Koppen–Geiger climate division, and is characterized by a temperate climate with hot and dry summers, but with rainy winters [29,54,55]. In 2017, between April and December, the Portuguese territory was under severe/extreme drought [29]. The average of the monthly precipitation from June to the end of September in the local of the orchard reached values below 30 mm (Figure 4A), which is typical of a dry summer according to Köppen climate classification [54,55]. Also, the orchard’s air temperature reached values around 38 °C in June, and even during October, the maximum air temperature reached values above 30 °C (Figure 4A). Two distinct adjacent areas of the orchard were selected and followed from June to the end of September, one without water supply, with dried topsoil and olive trees with leaves and fruits with visible symptoms of dehydration (dehydrated trees, dry area), and the other located near a groundwater supply providing constant water to olive trees (hydrated area). On the second day of October 2017, leaves (young and fully developed) from five trees of each area (Figure 4B,C) were sampled from different parts of the trees, frozen in liquid nitrogen, and then stored at −80 °C.

### 4.3. Determination of the Tree Water Status

Tree water status was evaluated by measuring the leaf water content. Leaf fresh weight was measured, and then the leaf was dried at 40 °C until constant weight. The leaf water content (LWC, %) was calculated as ((fresh mass-dry mass)/fresh mass) × 100.

### 4.4. Malondialdehyde (MDA) Determination

To quantify the MDA [56], the powder of frozen leaves (100 mg) was homogenized with 1.5 mL of 0.1% (*w*/*v*) trichloroacetic acid (TCA) and centrifuged for 10 min at 10,000× *g*, 4 °C. Two aliquots containing 250 µL of supernatant were collected. One aliquot was mixed with 1 mL of 20% (*w*/*v*) TCA, and to the other one with 1 mL of 20% (*w*/*v*) trichloroacetic acid and 0.5% (*w*/*v*) thiobarbituric acid (TCA + TBA). Both aliquots were placed in a hot bath (95 °C) for 30 min, cooled, and centrifuged at 10,000× *g* (10 min at 4 °C). Specific absorbance was measured at 532 nm and the non-specific absorbance at 600 nm. MDA was calculated by subtracting the non-specific absorbance from the specific absorbance and using the extinction coefficient of 155 mM^−1^ cm^−1^ [56]. Data are presented as mean ± standard deviation (*n* = 9, per condition).

### 4.5. Antioxidant Enzyme Analysis

Frozen olive leaf powder was homogenized with 0.1 M phosphate buffer (pH 7), 0.5 M Na_2_EDTA, 1% PVP (*m*/*v*), 1 mM PMSF, 0.2% Triton X-100 (*v*/*v*) and 2 mM DTT. The activity of the enzymes superoxide dismutase (SOD, EC 1.15.1.1), catalase (CAT, EC 1.11.1.6), ascorbate peroxidase (APX, EC 1.11.1.11), and guaiacol peroxidase (GPOX, EC 1.11.1.7) was measured in the supernatant. SOD was measured according to Agarwal et al. (2005) [57]. The reaction mixture contained 50 mM of potassium phosphate buffer (pH 7.8), 0.1 mM of EDTA, 50 mM Na_2_CO_3_, supernatant, 13 mM methionine, 63 mM nitroblue tetrazolium (NBT) and 2 µM riboflavin. The mixture was irradiated with fluorescent light (15 W) for 15 min. A non-irradiated mixture (blank) and a mixture without the illuminated supernatant (control) were prepared in parallel. The absorbance of the mixtures was read at 560 nm. One unit of SOD activity is the amount of enzyme necessary to inhibit the NBT reduction by 50% (per minute). To determine CAT activity [58], 500 µL of ultrapure water, 0.1 M potassium phosphate buffer (pH 7), 50 µL supernatant, and 6 mM H_2_O_2_ were mixed. After 5 min of the reaction starts, 1 mL of titanium reagent (1 g titanium dioxide and 10 g potassium sulfate were dissolved in 150 mL sulfuric acid) was added to stop the reaction. The mixture was centrifuged (10,000× *g*, 10 min at 4 °C), and the absorbance was read at 415 nm. CAT activity was determined using a calibration curve prepared with known concentration of catalase ($y=0.4639e^{-23.32x}$ and R^2^ = 0.98) [58]. The method described by Nakano and Asada (1981) [59] was used to measure the activity of APX. The reaction mixture contained 50 mM potassium phosphate buffer (pH 7.5), 0.1 mM EDTA, 0.5 mM ascorbic acid and supernatant. The reaction was started with 0.1 mM H_2_O_2_, and the reduction of ascorbate was followed at 290 nm. The activity of APX was calculated using the molar extinction coefficient of ascorbic acid (ε = 2.8 mM^−1^ cm^−1^). To measure GPOX activity, the method described by Pütter (1974) [60] was used. GPOX activity was measured by adding 100 mM phosphate buffer (pH 7), 15 mM guaiacol, and supernatant. The reaction was started with 3 mM of H_2_O_2_. The oxidation of guaiacol was followed at 470 nm, and the GPOX activity calculated using the tetraguaiacol molar extinction coefficient (ε = 26.6 mM^−1^ cm^−1^). Data are presented as mean ± standard deviation (*n* = 9, per condition).

### 4.6. Determination of Superoxide Radical (O_2_^•–^) and H_2_O_2_ Content

O_2_^•–^ content was measured according to Chaitanya and Naithani (1994) [61]. Briefly, leaves (100 mg) were incubated with 2 mL of extraction buffer containing 0.01 M phosphate buffer (pH 7.8), 0.05% NBT dissolved in 100 mL of DMSO, and 10 mM of sodium azide. Samples were incubated for 1 h at room temperature with agitation. The samples were then centrifuged for 2 min at 13,000× *g*, and 1.5 mL of supernatant was incubated at 85 °C for 15 min. After cooling, the absorbance was read at 580 nm. The content in H_2_O_2_ was measured as described by Zhou et al. (2006) [62]. Leaves (100 mg) were grounded in 1 mL precooled 5% TCA (*w*/*v*) and 0.15 g of activated charcoal. The mixture was centrifuged (12,000× *g*, 15 min at 4 °C), and then the pH of the supernatants adjusted to 8.4 with 17 M ammonia. The supernatant was divided into two aliquots, and 8 µg of catalase was added only to one aliquot. After 10 min at room temperature, the colorimetric reagent [10 mg of 4-aminoantipyrine, 10 mg of phenol, 5 mg of peroxidase (150 U mg^−1^) dissolved in 50 mL of 100 mM acetic acid buffer (pH 5.6)] was added to both aliquots. After 10 min at 30 °C, the absorbance was read at 505 nm. The amount of H_2_O_2_ was determined using a calibration curve prepared with known concentration of H_2_O_2_ (y=0.0004x+0.1148) and R^2^ = 0.99) [62]. Data are presented as mean ± standard deviation (*n* = 9, per condition).

### 4.7. Lipophilic Extract Preparation and Gas Chromatography–Mass Spectrometry (GC–MS) Analysis

Approximately 50 g (fresh weight) of leaf powder was mixed with 500 mL of *n*-hexane with stirring for 72 h and at room temperature. After the first extraction, the *n*-hexane was renewed, and an additional extraction in the same conditions was made. Then, the solvent evaporated to dryness with a rotary vacuum evaporator, and the extracts were used for the GC–MS analysis. For the silylation procedure, 200 µL of the extract solution (approximately 30 mg mL^−1^), 200 µL of internal standard (tetracosane, 0.46 mg mL^−1^), 50 µL of dichloromethane, 250 µL of pyridine, 250 µL of *N*,*O*-bis(trimethylsilyl) trifluoroacetamide and 50 µL of trimethylsilyl chloride were incubated in glass tubes in a water bath (70 °C) for 30 min [8]. Each silylated extract was injected in a GC–MS QP2010 Ultra Shimadzu equipped with a FactorFour Capillary Column VF-5ms 30 m × 0.25 mm ID DF = 0.25. The chromatographic conditions were as follows: carrier gas, He at a linear velocity of 40.0 cm s^−1^; column oven temperature, 70 °C; injection temperature, 320 °C; injection mode, split; pressure, 76.1 kPa; total flow, 63.7 mL min^−1^; column flow, 1.19 mL min^−1^; purge flow 3.0 mL min^−1^; split ratio, 1:50. The initial temperature was 70.0 °C for 5 min, followed by an increase of 4 °C up to 250 °C and then 2 °C up to 300 °C for 5 min. The total program time was 80 min. The mass spectrometer conditions: ion source temperature, 200 °C; interface temperature, 300 °C; solvent cut time, 6.5 min; micro scan width, 0 µs; threshold, 0; detector voltage, relative to the tuning result, 0.1 kV. The start time was 6.5 min and the end time at 80.0 min. The acquisition mode was scan, with event time at 0.10 s, the scan speed of 20,000, the start *m*/*z* at 50.0, and the end *m*/*z* at 1000.0. The compounds were identified based on a direct comparison with the mass spectra database’s libraries (NIST14 Mass spectral and WILEY Registry TM of Mass Spectra Data) and comparing the retention times and mass spectra of the standard compounds injected in the same chromatographic conditions of the samples. Calibration curves of pure compounds (cholesterol, maltose, octadecane, palmitic acid, and sorbitol) were used for the quantitative analysis. The correlation coefficient of the calibration curves was above 0.97. The pure compounds were injected (after silylation) in the same chromatographic conditions of the samples. At least four independent analyses were performed per condition.

### 4.8. Phenolic Extract Preparation and Ultra-High-Performance Liquid Chromatography–Mass Spectrometry (UHPLC–MS) Analysis

Approximately 50 g (fresh weight) of leaf powder was mixed with 500 mL of methanol with stirring for 72 h and at room temperature. After the first extraction, the solvent was renewed one more time. Each extraction solvent was evaporated to dryness with a rotary vacuum evaporator, and the extracts were used for the UHPLC–MS analysis. For the UHPLC–MS analysis, the final concentration of samples was 15 mg mL^−1^. Samples were filtered through a 0.2 µm Nylon membrane (Whatman, Buckinghamshire, UK) and injected in a UHPLC–MS (Thermo Scientific Ultimate 3000RSLC (Dionex, Sunnyvale, CA, USA)) equipped with a Dionex UltiMate 3000 RS diode array detector and coupled to a mass spectrometer [8]. The column used was a Thermo Scientific Hypersil Gold column (100 mm × 2.1 mm) with a particle size of 1.9 µm, and its temperature was maintained at 30 °C. The mobile phase was composed of (A) acetonitrile and (B) 0.1% formic acid (*v*/*v*), both degassed and filtered before use. The flow rate was 0.2 mL min^−1^. The solvent gradient was 5% of solvent B in the first 14 min, followed by 40% of solvent B for 2 min, 100% over 7 min, and 5% over 10 min. The injection volume was 2 µL. UV–V is spectral. Data were gathered in a range of 230 to 517 nm. The mass spectrometer used was an LTQ XL linear ion trap 2D equipped with an orthogonal electrospray ion source (ESI). The equipment was operated in negative-ion mode with an electrospray ionization source of 5.00 kV and ESI capillarity temperature of 275 °C. The full scan covered a mass range of 50–2000 *m*/*z*. Collision-induced dissociation MS/MS and MS^n^ experiments were simultaneously acquired for precursor ions. The chromatograms obtained at 280, 240, and 230 nm were analyzed, and the qualitative profile was performed. The extract’s phenolic compounds were identified based on UV–Vis spectra and MS^n^ spectra data with those closest available reference standards and data reported in the literature. The semi-quantification of the individual compounds in the plant extracts was performed by peak integration at 240 nm through the external standard method. The detection and quantification limits (LOD and LOQ, respectively) were determined from the calibration curves. The calibration curves were determined by injecting pure standard compounds (luteolin, oleuropein, and quercetin). These compounds’ concentrations guarantee each compound’s quantification in the extracts by interpolation in the calibration curve. The correlation coefficients confirmed the linearity of the calibration curves (R^2^ > 0.95). At least four independent analyses were performed per condition.

### 4.9. Statistical Analysis

The student’s *t*-test was used to evaluate the statistical significance between groups (hydrated and dehydrated). The significance level was 0.05. Pearson’s correlation was analyzed. All statistical analyses were performed in the SigmaStat program for Windows, version 3.1. (Systat Software, San Jose, CA, USA). Log_2_ fold change of two groups’ means was calculated using Microsoft^®^ Excel for Mac (version 16.32).

## 5. Conclusions

The environmental conditions of the summer of 2017 induced water deficit in olive trees of the dry area of the orchard. This lower leaf water availability was associated with the loss of long-chain alkanes and fatty acids levels, which were the main components or wax cuticles that protect from water loss. Moreover, the dry area trees showed high ROS levels (H_2_O_2_ and O_2_^•–^), but the olive antioxidant system was effective in controlling oxidative damages, preventing the augment of lipid peroxidation. This olive antioxidant response was mainly due to the upregulation of GPOX to reduce the H_2_O_2_ harmful effect with the complement of some flavonoids (luteolin-7-*O*-glucuronide), also acting as ROS scavengers. The dynamic response of some secoiridoids (e.g., 6’-*O*-[8-hydroxy-2,6-dimethyl-2-octenoyloxy] secologanoside and oleoside-11-methyl ester isomer decrease, and oleuropein and its aglycon increase) in trees of the dry area remains unclear, possible regulating stress response. This study demonstrates, for the first time, the leaf metabolomic adjustment in olive trees under a real scenario of field drought conditions. As reported in controlled environment experiments and extreme weather conditions, olive remarkable physiological plasticity was highlighted here. The high ability of physiological and metabolite adjustment under field stress will provide the necessary capacity to overcome and survive the extreme climatic conditions expected in the Mediterranean region in the following decades. The impacts of these weather conditions on fruits and oil quality are also a matter of concern and are under study.

## Figures and Tables

**Table 1:**
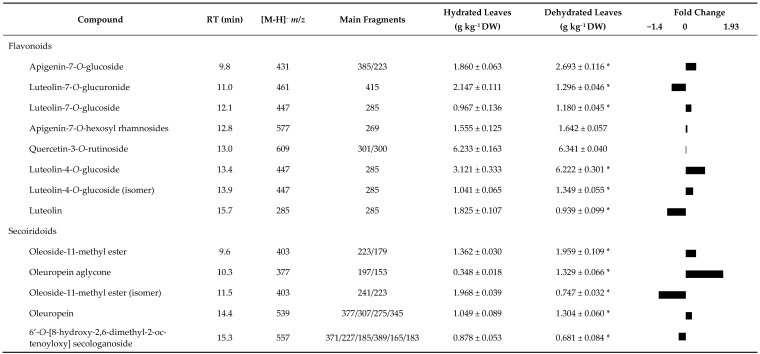
Phenolic profile of olive leaves from trees of the hydrated and dry areas.

Values are mean ± standard deviation (*n* *=* 4). Within the same line, the asterisk (*) indicates significant differences (*p* ≤ 0.05) between conditions. Fold changes (Log_2_ (dehydrated/hydrated leaves)) of metabolites in Cobrançosa leaves.

**Table 2:**
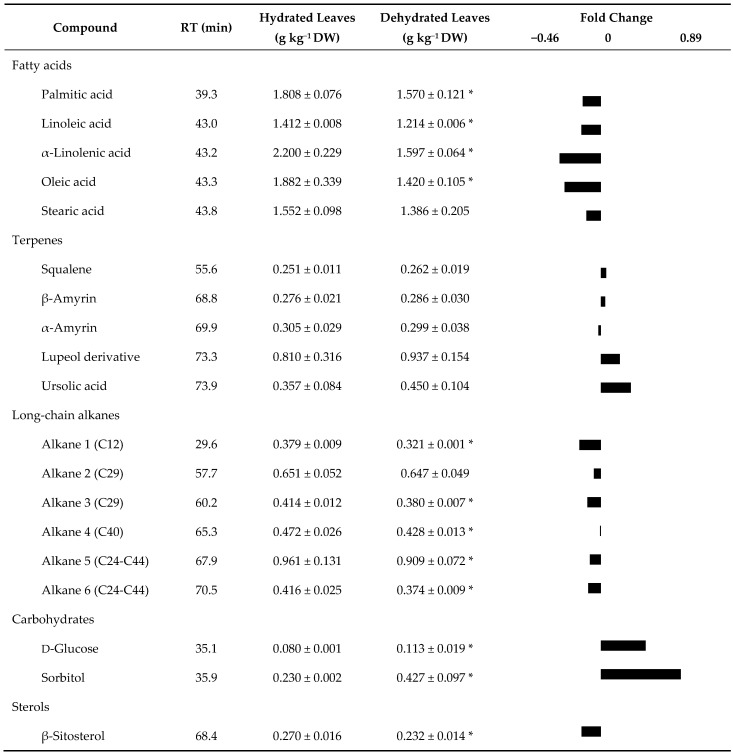
Lipophilic profile of olive leaves from trees of the hydrated and dry areas.

Values are mean ± standard deviation (*n* = 4). Within the same line, the asterisk (*) indicates significant differences (*p* ≤ 0.05) between conditions. Fold changes (Log_2_ (dehydrated/hydrated leaves)) of metabolites in Cobrançosa leaves.
